# Narrative structure of *A Song of Ice and Fire* creates a fictional world with realistic measures of social complexity

**DOI:** 10.1073/pnas.2006465117

**Published:** 2020-11-02

**Authors:** Thomas Gessey-Jones, Colm Connaughton, Robin Dunbar, Ralph Kenna, Pádraig MacCarron, Cathal O’Conchobhair, Joseph Yose

**Affiliations:** ^a^Fitzwilliam College, University of Cambridge, Cambridge CB3 0DG, United Kingdom;; ^b^Mathematics Institute, University of Warwick, Coventry CV4 7AL, United Kingdom;; ^c^London Mathematical Laboratory, London W6 8RH, United Kingdom;; ^d^Department of Experimental Psychology, University of Oxford, Oxford OX2 6GG, United Kingdom;; ^e^Centre for Fluid and Complex Systems, Coventry University, Coventry CV1 5FB, United Kingdom;; ^f^𝕃^4^ Collaboration, Institute for Condensed Matter Physics of the National Academy of Sciences of Ukraine, 79011 Lviv, Ukraine;; ^g^Mathematics Applications Consortium for Science and Industry, Department of Mathematics & Statistics, University of Limerick, Limerick V94 T9PX, Ireland;; ^h^Centre for Social Issues Research, University of Limerick, Limerick V94 T9PX, Ireland

**Keywords:** *A Song of Ice and Fire*, *Game of Thrones*, networks, Dunbar’s number, comparative literature

## Abstract

Network science and data analytics are used to quantify static and dynamic structures in George R. R. Martin’s epic novels, *A Song of Ice and Fire*, works noted for their scale and complexity. By tracking the network of character interactions as the story unfolds, it is found that structural properties remain approximately stable and comparable to real-world social networks. Furthermore, the degrees of the most connected characters reflect a cognitive limit on the number of concurrent social connections that humans tend to maintain. We also analyze the distribution of time intervals between significant deaths measured with respect to the in-story timeline. These are consistent with power-law distributions commonly found in interevent times for a range of nonviolent human activities in the real world. We propose that structural features in the narrative that are reflected in our actual social world help readers to follow and to relate to the story, despite its sprawling extent. It is also found that the distribution of intervals between significant deaths in chapters is different to that for the in-story timeline; it is geometric rather than power law. Geometric distributions are memoryless in that the time since the last death does not inform as to the time to the next. This provides measurable support for the widely held view that significant deaths in *A Song of Ice and Fire* are unpredictable chapter by chapter.

## Significance

We use mathematical and statistical methods to probe how a sprawling, dynamic, complex narrative of massive scale achieved broad accessibility and acclaim without surrendering to the need for reductionist simplifications. Subtle narrational tricks such as how natural social networks are mirrored and how significant events are scheduled are unveiled. The narrative network matches evolved cognitive abilities to enable complex messages be conveyed in accessible ways while story time and discourse time are carefully distinguished in ways matching theories of narratology. This marriage of science and humanities opens avenues to comparative literary studies. It provides quantitative support, for example, for the widespread view that deaths appear to be randomly distributed throughout the narrative even though, in fact, they are not.

The series *A Song of Ice and Fire* (hereinafter referred to as *Ice and Fire*) is a series of fantasy books written by George R. R. Martin. The first five books are *A Game of Thrones* (1), *A Clash of Kings* (2), *A Storm of Swords* (3), *A Feast for Crows* (4), and *A Dance with Dragons* (5). Since publication of the first book in 1996, the series has sold over 70 million units and has been translated into more than 45 languages. Martin, a novelist and experienced screenwriter, conceived the sprawling epic as an antithesis to the constraints of film and television budgets. Ironically, the success of his books attracted interest from film-makers and television executives worldwide, eventually leading to the television show *Game of Thrones*, which first aired in 2011.

Storytelling is an ancient art form which plays an important mechanism in social bonding (6–8). It is recognized that the social worlds created in narratives often adhere to a principle of minimal difference whereby social relationships reflect those in real life—even if set in a fantastical or improbable world (9). By implication, a social world in a narrative should be constructed in such a way that it can be followed cognitively (10). However, the role of the modern storyteller extends beyond the creation of a believable social network. As well as an engaging discourse, the manner in which the story is told is important, over and above a simple narration of a sequence of events. This distinction is rooted in theories of narratology advocated by coworkers Schklovsky and Propp (11) and developed by Metz, Chatman, Genette, and others (12–14).

Graph theory has been used to compare character networks to real social networks (15) in mythological (16), Shakespearean (17), and fictional literature (18). To investigate the success of *Ice and Fire*, we go beyond graph theory to explore cognitive accessibility as well as differences between how significant events are presented and how they unfold (19). A distinguishing feature of *Ice and Fire* is that character deaths are perceived by many readers as random and unpredictable. Whether you are ruler of the Seven Kingdoms, heir to an ancient dynasty, or Warden of the North, your end may be nearer than you think. Robert Baratheon met his while boar hunting, Viserys Targaryen while feasting, and Eddard Stark when confessing a crime in an attempt to protect his children. Indeed, “Much of the anticipation leading up to the final season (of the TV series) was about who would live or die, and whether the show would return to its signature habit of taking out major characters in shocking fashion” (20). Inspired by this feature, we are particularly interested in deaths as signature events in *Ice and Fire*, and therefore, we study intervals between them (21). To do this, we recognize an important distinction between story time and discourse time. Story time refers to the order and pace of events as they occurred in the fictional world. It is measured in days and months, albeit using the fictional Westerosi calendar in the case of *Ice and Fire*. Discourse time, on the other hand, refers to the order and pacing of events as experienced by the reader; it is measured in chapters and pages.

We find the social network portrayed is indeed similar to those of other social networks and remains, as presented, within our cognitive limit at any given stage. We also find that the order and pacing of deaths differ greatly between discourse time and story time. The discourse is presented in a way that appears more unpredictable than the underlying story; had it been told following Westerosi chronology, the perception of random and unpredictable deaths may be much less shocking (22, 23). We suggest that the remarkable juxtaposition of realism (verisimilitude), cognitive balance, and unpredictability is key to the success of the series.

## Materials and Methods

To perform this investigation we draw on two datasets. The first was extracted manually from *Ice and Fire* by carefully reading the text and noting interactions between characters. To facilitate comparisons to them, we follow methodologies developed for network analyses of medieval epics (16, 24–26) whereby characters are deemed to have interacted if they directly meet each other or it is explicitly clear from the text they knew one another, even if one or both are dead by that point in the story. (To our knowledge, no automated method currently exists that has been proven to match this manual approach; see, e.g., ref. 27.) From this dataset we construct a network of all of the characters in *Ice and Fire* who interact with at least one other. Characters are identified as nodes and interactions between them identified as edges (links). We also gathered temporal data on character deaths for interevent time analysis.

**Fig. 1.**
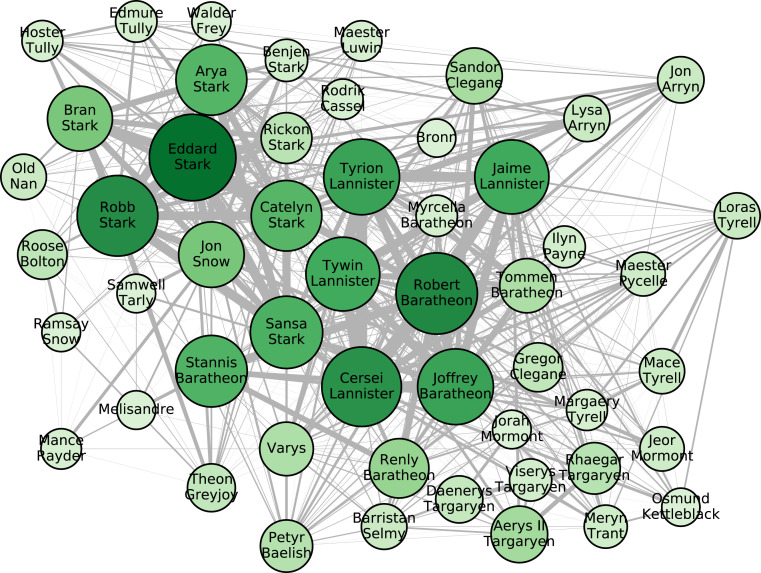
Network of the most predominant characters. For illustrative purposes we size nodes proportional to the number of chapters in which the characters interact. Edge thicknesses represent the numbers of times that corresponding pair of characters interact in the narrative.

Fig. 1 presents, for illustrative purposes, a subset of the network showing the most predominant characters. *SI Appendix* contains a similar figure showing only those characters still alive at the end of the fifth book (the survivor network). Predominance for these illustrations is measured by the number of chapters in which a given character interacts with at least one other character, and nodes are sized accordingly. Each character in Fig. 1 interacts in at least 40 chapters. The full network is far greater in extent. The thickness of the various edges represents the strength of links between nodes as the number of times the corresponding pair of characters interact in the narrative. Fig. 1 is therefore a visual representation of the primary characters and their interactions. For example, the enduring importance of characters such as Eddard Stark and Robert Baratheon is clear, despite the fact that both perished early in the story.

To analyze the dynamics and evolution of the narrative we also use a second dataset. This is an approximate timeline of the events of *Ice and Fire* indexed by the Westerosi calendar date, compiled by fans and followers, and maintained by the Reddit user identified as PrivateMajor (https://www.reddit.com/r/asoiaf/comments/1c07jw/spoilers_all_most_precise_asoiaf_timeline_in/). This timeline makes a number of assumptions which are noted in the dataset. Many of these dates are educated guesses because no explicit in-story timeline is provided by the author. According to the Reddit timeline, the opening events of *A Game of Thrones* take place on 22 April of the year 297, and the closing events of *A Dance with Dragons* take place on 8 February in the year 300. We used this second set of data to assign an approximate date to each chapter of each book, allowing us to study events as they occur within the in-story timeline. In many cases, chapters clearly span multiple days. In such cases we use the date corresponding to the earliest dated event occurring in that chapter. This allows us to order the data in two ways, the order in which the events happen (story time) and the way in which the narrative is told (discourse time).

There are multiple measures of network architecture, nodal importance, and edge weights. To address the primal issue of societal topology we analyze the full unweighted network. We assign a degree to each character as the number of connections it has to other nodes of the network, and we track average values over story and discourse time. Studies have shown that real social networks tend to have properties which distinguish them from other complex networks (15). Notable among these is homophily—the tendency of people to associate with people who are similar to themselves (28). One quantitative measure of homophily is assortativity, the extent to which the degrees of pairs of connected vertices are correlated (29). A network which has a positive correlation is called assortative, and one with a negative correlation is disassortative.

As degree measures how connected a node is, centrality quantifies how close it is to the core of the network. There are various measures, and common examples are betweenness, closeness, page rank, and eigenvector centrality. We use these tools holistically—no one tool gives a definitive characterization of verisimilitude or narratology, but together they build a picture that we can compare to real networks (15) and to mythological (16), Shakespearean (17), and fictional literature (18). In the next section we present betweenness, which is a normalized measure of the number of shortest paths (geodesics) between all other nodes that include the particular node in question (30). Nodes with high betweenness are important conduits for information transfer and in this sense tend to be more influential. Further details on data acquisition methodology, network construction, and analysis are provided in *SI Appendix*. The data and associated analysis codes are available in ref. 31.

## Results

*Ice and Fire* is presented from the personal perspectives of 24 point of view (POV) characters. A full list of them, ranked by the numbers of chapters from their perspectives, is provided in *SI Appendix*. Of these, we consider 14 to be major: eight or more chapters, mostly titled with their names, are relayed from their perspectives. Tyrion Lannister is major in this sense because the 47 chapters from his perspective are titled “Tyrion I,” “Tyrion II,” etc. Arys Oakheart does not meet this criterion as the only chapter related from his perspective is titled “The Soiled Knight.” We open this section by reporting how network measures reflect the POV structure. We then examine the network itself—how it evolves over discourse time, its verisimilitude, and the extent to which it is cognitively accessible. Finally, we analyze the distributions of time intervals between significant deaths and contrast these as measured in story time versus discourse time.

### Most Important Characters.

In networks, properties such as degree and centrality are signifiers of node importance. We now rank nodes according to these measures to examine the extent to which they correlate with the POV list.

Table 1 lists the 10 characters with the greatest degree and those with the greatest betweenness. We present results for the full network and the survivor network. The latter contains only those characters possibly still living by the end of the fifth book (e.g., a major character whose fate is uncertain by the end of *A Dance With Dragons* is treated as alive).

**Table 1.** Characters ranked by various network attributes

Degree	Betweenness centrality
Full network	
1. Jon Snow (214)	1. Jon Snow (0.0889)
2. Jaime Lannister (212)	2. Barristan Selmy (0.0831)
3. Tyrion Lannister (209)	3. Arya Stark (0.0777)
4. Catelyn Stark (204)	4. Tyrion Lannister (0.0700)
5. Arya Stark (192)	5. Theon Greyjoy (0.0671)
6. Theon Greyjoy (175)	6. Jaime Lannister (0.0606)
7. Cersei Lannister (161)	7. Catelyn Stark (0.0568)
**8. Robb Stark (158)**	**8. Stannis Baratheon (0.0519)**
9. Sansa Stark (156)	**9. Tywin Lannister (0.0356)**
10. Barristan Selmy (156)	10. Eddard Stark (0.0351)
12. Eddard Stark (140)	12. Sansa Stark (0.0275)
16. Brienne of Tarth (108)	13. Cersei Lannister (0.0250)
17. Bran Stark (106)	14. Brienne of Tarth (0.0236)
19. Daenerys Targaryen (104)	17. Samwell Tarly (0.0207)
20. Samwell Tarly (103)	18. Bran Stark (0.0202)
51. Davos Seaworth (72)	21. Daenerys Targaryen (0.0185)
	25. Davos Seaworth (0.0167)
Survivor network	
1. Tyrion Lannister (162)	1. Tyrion Lannister (0.0972)
2. Jon Snow (150)	2. Barristan Selmy (0.0952)
3. Jaime Lannister (149)	3. Arya Stark (0.0923)
4. Arya Stark (135)	4. Theon Greyjoy (0.0909)
5. Sansa Stark (122)	5. Jon Snow (0.0871)
6. Cersei Lannister (120)	**6. Stannis Baratheon (0.0812)**
7. Theon Greyjoy (115)	7. Jaime Lannister (0.0805)
8. Barristan Selmy (103)	8. Sansa Stark (0.0408)
**9. Stannis Baratheon (86)**	9. Samwell Tarly (0.0320)
10. Brienne of Tarth (83)	10. Cersei Lannister (0.0310)
12. Samwell Tarly (79)	12. Brienne of Tarth (0.0274)
18. Daenerys Targaryen (69)	13. Bran Stark (0.0248)
20. Bran Stark (68)	17. Davos Seaworth (0.0184)
38. Davos Seaworth (54)	33. Daenerys Targaryen (0.0093)

Characters are ranked by degree and betweenness centrality (with values in parentheses). The three non-POV characters that appear in the top 10 are highlighted in boldface, and major POV characters who do not appear in the top 10 are also listed. Qualitatively, it appears that the 14 major POV characters correlate well with the most important characters by both measures.

Table 1 also lists major POV characters that lie outside the top 10. Degree and betweenness are very different indicators of importance from the notion of POV characters. However, POV characters form the majority of the top 10 characters when ranked by either measure. There are only three non-POV characters in Table 1; Robb Stark, Stannis Baratheon, and Tywin Lannister. These are highlighted in bold type. The effectiveness of network measures at qualifying character importance is established by the fact that both rankings primarily pick out the POV characters. Here we use betweenness as indicative of centrality with other measures presented in *SI Appendix*. Different centrality measures paint similar pictures, suggesting the importance portraits they deliver are quite robust in network terms.

### Evolution of the Social Network Structure.

**Fig. 2.**
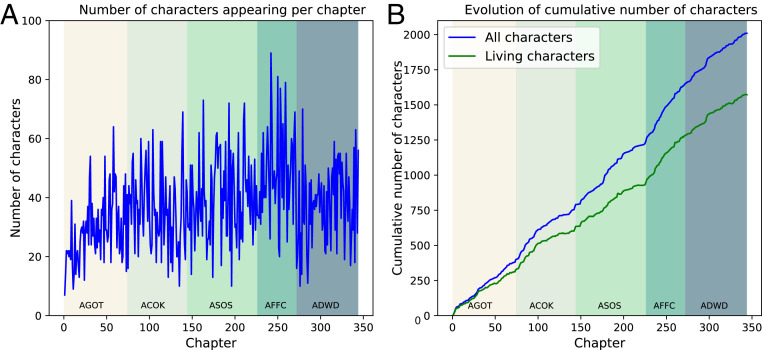
Number of characters in the narrative. (*A*) Number of characters appearing in each individual chapter. This shows significant fluctuations chapter by chapter and fluctuates around 35 by the end of *A Game of Thrones*. (*B*) Evolution of the cumulative number of characters appearing in the narrative by chapter (blue) and of characters introduced who have not yet died (green). Both curves grow approximately linearly throughout *Ice and Fire*. Labels AGOT, ACOK, ASOS, AFFC, and ADWD represent *A Game of Thrones*, *A Clash of Kings*, *A Storm of Swords*, *A Feast for Crows*, and *A Dance with Dragons*, respectively.

From the five books containing 343 chapters, 2,007 characters were identified, of which 1,806 interact with another at least once. Fig. 2 depicts how character numbers evolve as the discourse unfolds. The numbers of characters appearing in each individual chapter are plotted in Fig. 2*A*. These numbers range from 7 for the first chapter up to 89 for chapter 16 of *A Feast for Crows*. After a short period of growth in the first book, in which the main characters are introduced, the number of characters per chapter settles at around 35. This value has been identified as a stable subgrouping within social networks (32) and as the typical size of (contemporary) bands of hunter-gatherers (33). It has also been identified as the typical cast size in Shakespeare’s plays (17) and optimal size for English language and literature research centers (34, 35). The cumulative number of characters (introduced up to and including a given chapter) is plotted in blue in Fig. 2*B*. Those who have not explicitly died by the fifth book are depicted in green. The near linear growth of each curve indicates remarkable stability throughout the series.

**Fig. 3.**
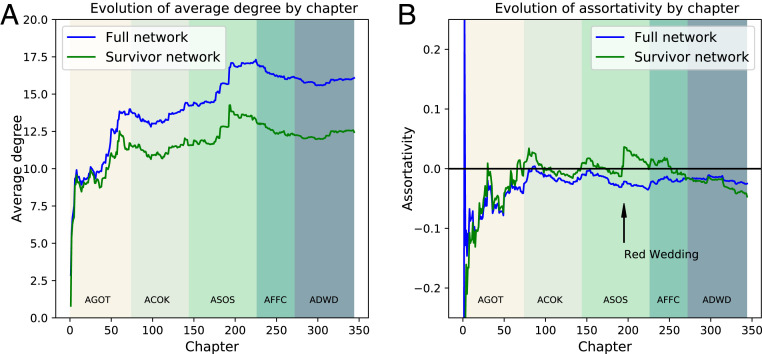
Evolution of network properties by chapter labeled as in Fig. 2. (*A*) Evolution of the average degree. After a period of initial growth as main characters are introduced, the average degree stabilizes at around 16 for the network involving all characters and around 12 if only the living characters are included. (*B*) Evolution of the degree assortativity. After the first book (*A Game of Thrones*), the assortativity for the living-character network fluctuates around 0. While the assortativity of the full network (blue) also fluctuates, it stays slightly disassortative for the later books.

Fig. 3*A* (which has the same color scheme as Fig. 2) depicts the chapter-by-chapter evolution of the mean degree, and Fig. 3*B* is the counterpart plot for assortativity. The average degree centers around 16 for the full network and around 12 for the survivor network, values that approximate the 15-layer in egocentric social networks (36–38). Although the average degree is small, the distribution is highly skewed as is common in social networks. When real social networks are constructed from data, one does not expect every low-degree node to necessarily have few connections due to sampling bias. The same applies to our fictional social network; since the the narrative is relayed from individual perspectives, the ego networks of POV characters feature more than those of less prominent characters. For example, the highest degree value of 214 belongs to POV character Jon Snow and contrasts markedly with 214 characters having degree 1. The 14 major POV characters have an average degree of 154.0 within the network of all characters, with SD 47.0. This is close to Dunbar’s number of 150, the average number of stable relationships usually maintained at any given point in human life (33).

Another consequence of the POV style is the suppression of assortativity compared to real social networks after the fourth book. The deflated degrees of the masses relative to POV characters decrease homophily. The corollary of this effect is visible in the survivor assortativity jump seen in the third book when Catelyn Stark, an important POV character, is murdered along with some other notable characters at the Red Wedding. After an initial growth period in the first book, assortativity fluctuates around 0, before dropping to a slightly negative value (−0.03) by the fifth book. This is lower than most values measured in real social networks (15) but not by much. It is certainly sufficient to endow *Ice and Fire* with a greater degree of verisimilitude than more egocentric networks such as *Beowulf* or the *Táin Bó Cuailgne* (16). In comparative mythological terms, *Ice and Fire* has a narrative networks more akin to those of the Icelandic sagas (24).

Therefore, despite the continuous introduction of new characters, the author has managed to maintain a consistent social network structure. The number of these interactions is at the upper end of the cognitive capacity of an average reader. Hence, while there is a vast number of characters and even greater number of interactions in *Ice and Fire* at any given stage of the narrative, the social network a reader has to consider in order to follow the story is similar in scale to natural cognitive capacity.

### Distributions of Interevent Times for Significant Deaths.

**Fig. 4.**
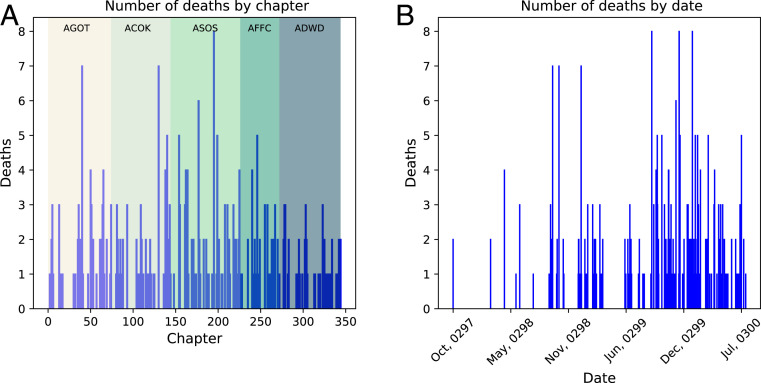
Timeline of significant character deaths in *Ice and Fire*. (*A*) Number of deaths by chapter (discourse time). (*B*) Number of deaths by date (story time).

We now turn to consider interdeath story time and interdeath discourse time to reveal an interesting difference between the underlying chronology and how the narrative is presented. For this purpose we consider only deaths which we deem to be significant. These are deaths of characters in the network who appear in more then one chapter. We apply this criterion to avoid the inclusion of the deaths of “cannon-fodder” characters whose main purpose in the story is to die immediately after they are introduced. Fig. 4*A* shows the number of significant character deaths by chapter (discourse time). Fig. 4*B* gives the same data ordered by date (story time). It is striking how deaths appear far more clumped together in story time than in discourse time. The structure of Fig. 4*A* helps explain the perception that death can occur unpredictably in the narrative, while Fig. 4*B* suggests extended safe periods where no deaths occur.

These observations can be quantified by examining the empirical distributions of the time intervals between deaths. Our data analysis follows the reasoning described in ref. 39, and all computations are performed using the associated R package (40). To explore the (un)predictability of *Ice and Fire* timelines, we consider the conditional probability that the number of steps (chapters or days) to the next event exceeds n+m given that it has already exceeded m. If this is the same as the unconditional probability that the waiting time exceeds n,

P(X>n+m | X>m)=P(X>n), [1]

then the interevent time distribution is said to be memoryless. In other words, knowing the time since the last event provides no information about the time to the next event. It is well known that the geometric distribution

$$P\left(X=n\right)\equiv P_{X}\left(n\right)=q\;{\left(1-q\right)}^{n-1},$$ [2]

is the only discrete interevent-time distribution that is memoryless and thus maximally unpredictable. Here q is a parameter to be fitted from data. Further details can be found in *SI Appendix*.

**Fig. 5.**
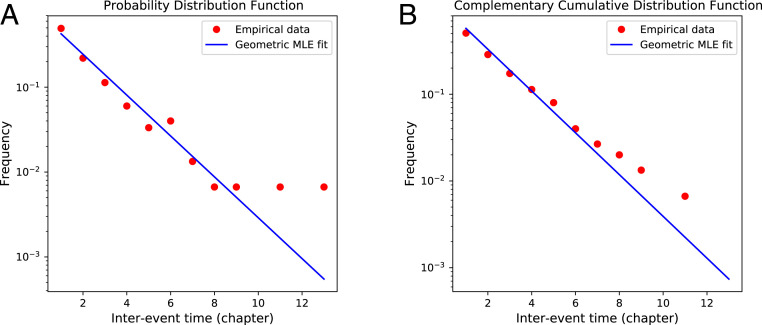
Empirical distributions of interevent times for significant deaths measured by chapter (discourse time), with fit to geometric distribution. A geometric distribution is memoryless in that it is what would be expected if deaths are maximally unpredictable throughout, as is suggested by many readers/viewers of the series.

Interevent discourse time data are presented in Fig. 5*A*, and the corresponding cumulative data are presented in Fig. 5*B*. We use the maximum likelihood method to determine the best fits to the geometric distribution for the data, and these are also plotted in Fig. 5. The associated *P* values characterize goodness of fit; we reject the hypothesis if the *P* value is less than 0.05. The results are as follows: discourse time, q=0.58, [0.50,0.68], *P* = 0.087; story time, q=0.12, [0.10,0.15], *P* ≈0.

**Fig. 6.**
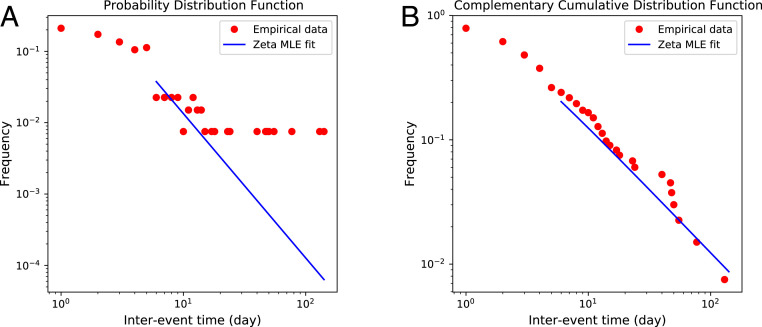
Empirical distributions of interevent times for significant deaths by date (story time). Date here is measured using the fictional Westerosi calendar. Shown in blue is the best-fit discrete power law (Zeta) distribution.

Here parenthesized values indicate the approximate 95% confidence intervals determined by bootstraping. These results suggest that interevent times for significant deaths in discourse time (chapters) are well described by a geometric distribution and are therefore memoryless. In contrast, the null hypothesis that significant deaths in story time (calendar) follow a memoryless geometric distribution can be rejected. These data are presented in Fig. 6.

Since events in story time are inconsistent with memorylessness, we consider an alternative to the geometric distribution. Evidence suggests that interevent time distributions for many (nonviolent) human activities in the real world, including communication, entertainment, trading, and work, have power-law tails, usually with exponents between 1 and 2 (39, 41, 42). Similar heavy tails have been observed in interviolence intervals (43, 44) as well as in human behavior in virtual environments (45). Therefore, we fit to a discrete power-law distribution of the form

$$P\left(X=k\right)\equiv P_{X}\left(k\right)=\frac{k^{-\alpha }}{\zeta \left(\alpha \right)}.$$ [3]

Here the exponent α controls the power law, and ζ(α) is the Riemann zeta function. The results are as follows: discourse time, α=3.9, [2.0,8.9],$x_{0}=3.7$, [ 1,7], *P* = 0.392; story time, α=2.00, [ 1.75,2.36],$x_{0}=3.6$, [2,8] , *P* ≈0.428.

Again, the uncertainties in parameters indicate approximate 95% confidence intervals and are estimated by bootstrapping. The fits, which are plotted for story time in Fig. 6, suggests that interevent times for significant deaths by date are indeed well described by a power-law distribution with a lower cutoff. Interestingly, the story time exponent α≈2 is comparable to the values seen in real-world human activities (39, 41–45), providing another sense in which the fictitious world of *Ice and Fire* bears quantitative similarity to the real social world.

At first sight the results for discourse time appear also to suggest that we cannot reject the hypothesis that interevent times for significant deaths by chapter can be matched by a power law distribution. However, the cutoff $x_{0}$ excludes long waiting times from the fit. Unlike the the single-parameter geometric fit, a power law does not match the entire range of the data. (The poorer match of the cutoff power law is also reflected in the uncertainties in the α parameter value which are much higher than their discourse time counterparts.) The memoryless geometric model is therefore the superior description of interevent times in discourse.

In summary, the interevent time distribution for significant deaths by discourse time is well fitted by a geometric distribution indicating that such events can seem to the reader to occur almost at random intervals. However, when analyzing deaths in terms of story time, this is not the case, with significant events occurring in a more natural way. Portraying significant events by discourse time instead of as they happen appears to maintain the reader’s suspense.

## Discussion

*A Song of Ice and Fire* is a prodigious modern epic of considerable complexity that remains accessible to a vast congregation of devotees. Among its appeals are the uncertainty and unpredictability of its storyline as characters, including important ones, can be killed off seemingly at random. Indeed, not even the POV characters are guaranteed safe passage from one book to the next. Here we have shown that the network properties of the society described in *Ice and Fire* are close to what we expect in real social networks (16). Also, by relating the story from the points of view of different characters, the total number of interactions at any given stage remains within the average reader’s cognitive limit, making it possible to keep track of these relationships (19).

The positioning of this paper relative to the context, initiatives, and aspirations of digital humanities merits further comment. The recent review (46) identified some of its methods and themes, developed in the context of comparative mythology and traditional epic narrative cycles in particular (8), as one of four focal points in the extraction and analysis of character networks (the remaining three foci being literary analysis, video narratives, and computer science methods aimed at data extraction). Here we go beyond such character network considerations by introducing two elements to quantitative narratology, namely, the questions of cognitive limits and the interplay between story time and discourse time (10). Unlike historical, quasi-historical, or mythological chronicles of societies and events, a key requirement for fictional storytelling in *Ice and Fire* is that it not spin out of control because of its enormous scale. Fictional narratives require widespread engagement for commercial success. Whatever the storyteller’s cognitive competences may be (7), he has to avoid overtaxing his reader’s ability to keep track of the action—itself related to the number of characters involved (10). If the story is allowed to become too complex, there is a threat of the average reader becoming cognitively overwhelmed and the story becoming chaotic and unfathomable (47). *Ice and Fire* avoids this; although more than 2,000 characters appear, readers and TV audiences alike remain avidly engaged.

The findings reported here suggest that this is facilitated by clever structuring such that each chapter is told by different POV characters, endowed with social networks containing only around 150 individuals. Moreover, there are only 14 major POV characters. These are frequent numbers in the structure of real social networks (32–34, 36–38, 48) and they allow the reader to work within natural templates; the story reflects experiences in the everyday social world and therefore does not overtax cognitive abilities that are evolved to match these scales (10).

Our findings on the constraints on the size and structure of the cast of characters are not peculiar to this particular drama. Similar numerical constraints have been reported for Shakespeare’s plays (17) and contemporary films (49). Much of this seems to reflect natural limits on mentalizing competences—the cognitive skills that underpin our ability to handle social relationships in the virtual mental sphere of the everyday social world (7, 10). These are limited to five orders of intentionality and provide the base from which the scaled layers of social networks are built up (7); more importantly, neuroimaging studies have shown that competences in this respect correlate directly with the number of individuals in the 15-layer (50, 51). That this is important for storytelling has been demonstrated by a series of experimental studies showing that enjoyment of a story is greatest when the number of levels of mentalizing (effectively the number of characters involved in a scene) is closest to the reader’s own mentalizing abilities (52). Krems and Dunbar (49) showed that Shakespeare, at least, seemed to adjust the number of characters in a scene to the effect of remaining within the mentalizing capacities of the audience.

Also, the characteristic unpredictability of the narrative appears in discourse time only, with associated interevent times for significant deaths well described by a memoryless distribution. In story time the plot unfolds in an altogether different manner for, chronologically, many characters die in a way consistent with regular human activities. The difference suggests that the author structures the order and pacing of significant events (consciously or subconsciously) to make the series more unpredictable. The distinction between story time and discourse time was first identified in the early part of the 20th century by the influential Russian formalist literary theorists, notably coworkers Schklovsky and Propp (11). Their distinction between fabula (story, or chronological sequence of events) and sjuzhet (plot) is essentially that which we draw here. Schklovsky, in particular, emphasized the importance of defamiliarization (decoupling the temporal sequence of the plot from the chronological storyline) as a device for engaging the reader in the story. Our analysis of significant deaths highlights how effectively Martin exploits this technique. However, the question of whether the quantitative distinction between story time and discourse time applies to less significant events remains open.

Thus, two important, but conflicting, requirements of effective storytelling are successfully married in *Ice and Fire*: 1) the reader’s attention is maintained by the unexpected sequencing of significant events to encourage page turning to find out why something happened or what happens next and 2) the reader’s sense of what is natural is not overtaxed (i.e., seemingly random events make sense). This remarkable marriage of verisimilitude (realism) and unpredictability (memorylessness) is achieved in a cognitively engaging manner.

In summary, we show that despite its massive scale, *Ice and Fire* is very carefully structured so as not to exceed the natural cognitive capacities of a wide readership. Despite its dynamic, extended temporal basis, the structure of its social world mirrors that of natural social networks in ways likely to minimize the cognitive burden on the reader. At the same time, the storyteller has manipulated the timeline of the story in such a way as to make it continuously more appealing by making significant events seem random so as to heighten the reader’s engagement. The identification of patterns of verisimilitude, cognition, and unpredictability through computational methods may inspire wider quantitative approaches to other areas of literary study, including drama, television, film, periodicity, genre, canonicity, literature, history, and fantasy.

## Supplementary Material

Supplementary File

## Data Availability

Text and code have been deposited in GitHub, https://github.com/colm-connaughton/ASOIAF-data-and_codes.
