# Supplementary material for: Narrative structure of A Song of Ice and Fire creates a fictional world with realistic measures of social complexity
Source: Proc Natl Acad Sci U S A. 2020 Nov 2;117(46):28582–8. doi: 10.1073/pnas.2006465117 (PMC7682562; doi:10.1073/pnas.2006465117)
Supplement: Supplementary File [file pnas.2006465117.sapp.pdf]

PNAS
www.pnas.org

Supplementary Information for

Narrative structure of A Song of Ice and Fire creates a fictional world with realistic measures of social complexity

Thomas Gessey-Jones, Colm Connaughton, Robin Dunbar, Ralph Kenna, Pádraig MacCarron, Cathal O'Conchobhair, Joseph Yose

Ralph Kenna.
E-mail: Ralph.Kenna@coventry.ac.uk

This PDF file includes:

Supplementary text
Figs. S1 to S2
Tables S1 to S2
SI References

www.pnas.org/cgi/doi/10.1073/pnas.2006465117

Supporting Information Text

Data acquisition and network construction

A. Discourse-time data. The study reported in the main text is of the first five books of *A Song of Ice and Fire*. A sixth book, *The Winds of Winter*, is currently being written (1) and a seventh, titled *A Dream of Spring*, is expected to be the concluding volume. Network data were extracted manually by one of us (O'Conchobhair) carefully reading and annotating the books over several months. Data were entered into a set of spreadsheets, one for each book. Each spreadsheet was broken down by chapters. As characters appear within each chapter, new rows were added to the spreadsheet and new columns were added containing the names of characters that they interact with. When a character appears for the first time in the entire series, this appearance is noted as a "debut" in a separate column. If a character dies in a given chapter, this appearance is noted as a "death" in a separate column. Interactions are deemed meaningful when two characters directly meet or when it is clear from the text that they knew each other, even if one or both is now dead. Judgement is required to determine when a given interaction is meaningful. The impossibility of harvesting all meaningful interactions using Natural Language Processing tools is why we prefer a manual data collection protocol over less time-consuming automated approaches.

Additional details on the initial data acquisition process include:

- Characters that are not named are not included except in exceptional circumstances when it is clear that namelessness follows from the plot (e.g. the "White Walkers", the "Faceless Men") rather than indicating inconsequentiality.
- We include characters and interactions that are mentioned in the recollections or stories of others - what we call "historical characters". Most historical characters predate the first book. The debut and death of such characters are recorded as the same chapter in which they are first mentioned.
- Multiple instances of a pair of characters interacting within a given chapter are treated as a single entry.
- We do not double-count interactions: if character A is recorded as having interacted with character B in a given chapter, we do not record a separate interaction if character B subsequently interacts with character A.

The data sets were then subject to post-processing to correct errors and ensure consistency. In cases of doubts regarding the data four other team members were consulted (Connaughton, Gessey-Jones, MacCarron and Yose) and opinions calibrated through discussion. Consensus assured that no automated calibration method was required (2). Post-processing involved the following checks:

- Standardization of spelling, capitalization and use of white spaces in character names.
- Removal of any unintended "blank" entries.
- Correction of any double-counted interactions.
- Accounting for aliasing. For example, Arstan Whitebeard appears in *A Clash of Kings* and, over time, the reader learns that this character is none other than Barristan Selmy, a noble Westerosi knight and former leader of the esteemed Kingsguard. Because they are one and the same, Arstan Whitebeard and Barristan Selmy are treated as a single individual.

B. Story-time data. The data sets described above are indexed by chapter and are readily used to measure the evolution of the narrative in discourse time. To generate the timeline of events in story time, a second data set is used. This was compiled by fans and followers of the series and is maintained by the Reddit user identified as PrivateMajor (3). It assigns dates in the Westerosi calendar to the main events in the narrative. Some of these dates are necessarily educated guesses because no explicit in-story timeline is provided by the author. We link the two data sets at chapter level. I.e., we assign all the events described in each chapter to a single date that is intended to be representative of that chapter. This means that the granularity of the temporal data extends to chapter level.

C. Network construction. The analysis contained in the main text is restricted to characters who interact with at least one other character. This criteria select 1806 characters from the 2007 unique characters contained in the original data. The criterion was chosen to minimise the fragmentation of the network. This is important when we calculate various centrality measures since quantities like betweenness, closeness and eigenvector centrality are only meaningfully applicable to the largest connected component of a network. It also goes some way to excluding historic characters, who are not involved in the story other then being briefly mentioned.

D. Reproducibility. The data and the codes used to generate the analyses, tables and figures contained in this paper are publicly available at (4).

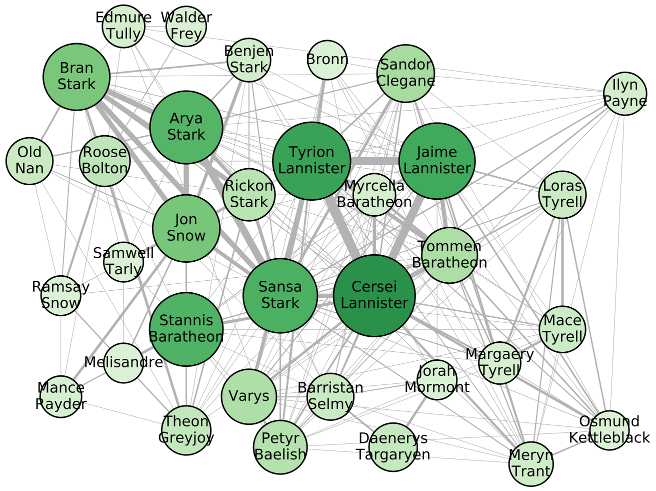

Fig. S1. Visualisation of the core of the survivor network, i.e., the characters still alive at the end of the fifth book. This sub-network contains the characters who interact with an other in at least 40 chapters, the complete network is much larger. Node size is proportional to the number of chapters in which a given characters interacts with another. Edge thickness is proportional to the number of times that a given pair of characters interact in the narrative.

Additional network visualisations

We include here some additional network visualisations for completeness.

Figure S1 shows the most predominant nodes in the survivor network - characters who have not explicitly died by the end of the fifth book. It is the survivor counterpart of Figure 1 of the main text, which includes predominant characters from the full network. As in the main text we determine predominance by the number of chapters in which the character interacts with an other.

Both networks are qualitatively similar. Together they illustrate the enduring importance of characters such as Eddard Stark and Robert Baratheon to the narrative despite the fact that both characters died in the first book.

The degree distributions of the full network and the survivor network are compared in Fig. S2. The two are qualitatively similar with a small numbers of very high degree nodes in the tails of both distributions.

Memoryless distributions

We provide here a summary of the mathematics underlying the notion of *memorylessness* of inter-event time distributions. A distribution of inter-event times is memoryless if knowing the time since the last observed event neither increases nor decreases the probability of observing an event in a future time interval. These results are well known to statisticians and probability theorists (see, for example, (5)) and are reproduced here solely to render the manuscript self-contained. We first describe the continuous case where times between events are real numbers. In this case, memorylessness is modelled by the exponential distribution. We then consider the discrete case where times between events are measured in integer units. In this case, the memorylessness comes from the geometric distribution, the discrete analogue of an exponential distribution.

Let X be a random variable representing the time between discrete events. In our case, events are the significant deaths of characters in *Ice and Fire*. If X is exponentially distributed, its probability distribution function is

$$\mathbb{P}(X = x) \equiv P_X(x) = \lambda\,\mathrm{e}^{-\lambda\,x}, \quad [1]$$

where λ is the rate parameter. It is the average rate at which events occur, since the mean waiting time is $\mathbb{E}[X] = 1/\lambda$. Although there is a well-defined mean waiting time, the distribution function is *memoryless* in the sense that knowledge of the time since the last event provides no information about the waiting time to the next event. To see this, we introduce the cumulative distribution function

$$\mathbb{P}(X \leq x) \equiv F_X(x) = 1 - \mathrm{e}^{-\lambda\,x}, \quad [2]$$

and its complement, the complementary cumulative distribution function (or survival function),

$$\mathbb{P}(X > x) \equiv S_X(x) = \mathrm{e}^{-\lambda\,x}. \quad [3]$$

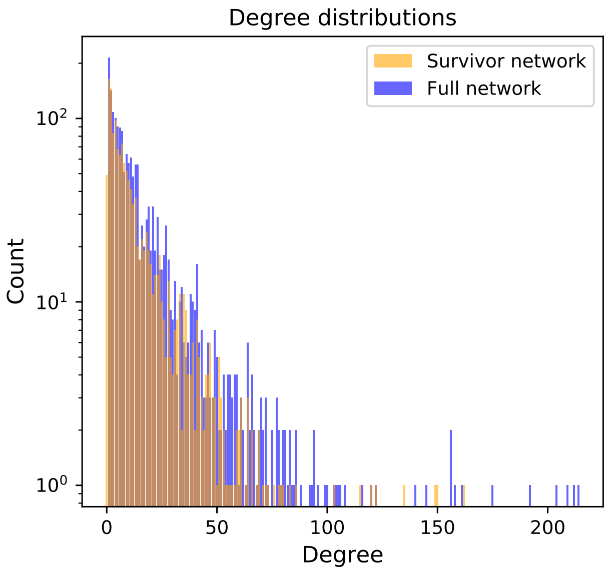

Fig. S2. Degree distribution for the full network of characters and the survivor network. Here Count is the number of characters with a given degree value. In the full network 214 characters have degree value one and POV character Jon Snow has the highest degree value of 214.

Using the law of conditional probability and Eq. (3), the left-hand side of Eq. (1) becomes

$$\begin{aligned}
\mathbb{P}(X > t+s \,|\, X > t) &= \frac{\mathbb{P}(X > t+s)}{\mathbb{P}(X > t)} \\
&= \frac{S_X(t+s)}{S_X(t)} \\
&= \frac{\mathrm{e}^{-\lambda\,(t+s)}}{\mathrm{e}^{-\lambda\,t}} \\
&= \mathrm{e}^{-\lambda\,s} \\
&= S_X(s) \\
&= \mathbb{P}(X > s).
\end{aligned}$$

This means that the probability that the waiting time exceeds $t+s$, given that it has already exceeded time t, is the same as the unconditional probability that the waiting time exceeds s. In other words, knowing that the event has not occurred for a time t neither increases nor decreases the probability that one will wait a further time s before the next event. Exponentially distributed waiting times are thus maximally unpredictable in the sense that knowing the time since the last event provides no information about the time to the next event.

However, the exponential distribution is continuous and waiting times between significant events in the narrative take integer values — chapters or days. We therefore consider the discrete analogue of the exponential distribution, namely the geometric distribution. This is the distribution of the number, X, of Bernoulli trials needed to deliver one event where the probability of success in a single trial is q. This distribution is supported on the positive integers. The corresponding probability distribution function, cumulative probability distribution function and complementary cumulative distribution function are given respectively by:

$$\mathbb{P}(X = n) \equiv P_X(n) = q\,(1-q)^{n-1}, \quad [4]$$

$$\mathbb{P}(X \leq n) \equiv F_X(n) = 1-(1-q)^{n}, \quad [5]$$

$$\mathbb{P}(X > n) \equiv S_X(n) = (1-q)^{n}. \quad [6]$$

A similar calculation to the one above illustrates that the geometric distribution also has the memoryless property, Eq. (1). Indeed, the exponential and geometric distributions are the only distributions with this property and they share a single functional form through the relation $\lambda = -\log(1-q)$.

Point of view (POV) characters

In Table S1 we present the POV characters ranked according to the number of chapters narrated from their perspectives. This list *excludes* prologues and epilogues which are usually narrated from the perspective of an insignificant character who often dies at the end of their chapter. We draw a distinction between major and minor POV characters. The former are named in most of their chapter title and have at least 8 chapters attributed to them. The latter are rarely named in their chapter titles, instead often referred to by descriptors, and are associated with fewer chapters - 4 at most. Independently of chapter titles, the gap between 4 and 8 chapters also makes this distinction plausible on numerical grounds.

Different network measures of character centrality

In Table S2 we compare the most highly ranked characters in *Ice and Fire* according to several centrality measures:

- Betweenness centrality (6): the normalized number of shortest paths between pairs of nodes that pass through a given node.
- Closeness centrality (6, 7): the reciprocal of the average distance of a node from all the other nodes on the graph.
- Eigenvector centrality (8, 9): a measure of relative importance where the importance of a node is the average of the importance of its immediate neighbours. A node with high eigenvector centrality is connected to many nodes which themeselves have high eigenvector centrality.
- Page rank (9, 10): a ranking based on the proportion of time a random walker on the graph spends visiting any given node. Mathematically this is a variation of eigenvector centrality in which the total contribution of any node to the scores of its neighbours is 1, regardless of how many neighbours that node has.

In Table S2 we list the top 15 characters by each of these measures of importance for both the full network (upper sub-table) and the survivor network (lower sub-table). Non-POV characters that appear in the top 15 are indicated in boldface. The ranks of any major POV characters that do not appear in the top 15 are also shown below the line in each case. We can draw several conclusions from these rankings

- With the exception of eigenvector centrality, each of these choices of centrality picks out comparable rankings of important characters, with the POV characters heavily represented. This indicates that our analysis is robust.
- The character Daenerys Targaryen stands out by how low her ranking appears in terms of betweenness and centrality. This is the case despite her being a central character to the story and major POV character. It reflects an important aspect of the plot: for all of the first five books, Daenerys spends her time on a different continent, far from most of the rest of the action. She is distanced from the other main characters both in network terms and in terms of plot.
- The distinctiveness of the rankings by eigenvector centrality is striking and reflects an interesting intersection of a feature of the plot with a feature of the algorithm. Note how the list of highest eigenvector centrality characters in the survivor network is dominated by members of House Frey. Walder Frey, the head of House Frey, is an important villain in the narrative, noted for his fecundity and his devastating betrayal of House Stark at the Red Wedding. His many offspring form a large, highly interconnected clique within the character network. This structure results in high eigenvector centrality; the best way to increase eigenvector centrality is to form many reciprocal links within a small group since this both increases the number of links to a node and increases the relative number of links of these neighbours. Interestingly, this ability to “game" the eigenvector score by cross-linking is one of the key problems that was addressed by Brin and Page in designing the Page Rank algorithm for ranking web pages that led to the creation of the Google search engine.
- We note that the page rank measure correlates most closely with the POV characters in both the full and survivor networks. Page Rank is not skewed by the strong cross-linking of Frey clique because the total contribution of a node to the score of its neighbours is always one: a “one node - one vote” model. Excessive cross-linking within a clique therefore only serves to dilute the score. Fans of the narrative may find it interesting to note that the Page Rank score is also the only one to identify the importance of Daenerys Targaryen.

References

1. GR Martin, Excerpt from the winds of winter (online at http://www.georgerrmartin.com/excerpt-from-the-winds-of-winter/) (2016).
2. R MacKay, R Kenna, R. Low, S Parker, Calibration with confidence: a principled method for panel assessment. Royal Soc. Open Sci. **4**, 160760 (2017).
3. u/PrivateMajor, Reddit: Most precise asoiaf timeline in existence (2013) Online at https://www.reddit.com/r/asoiaf/comments/1c07jw/spoilers_all_most_precise_asoiaf_timeline_in/.
4. T Gessey-Jones, , et al., Network of Thrones: Social networks and inter-event times in A Song of Ice and Fire: data and codes (online at https://github.com/colm-connaughton/ASOIAF-data-and_codes/releases/tag/v1.0) (2020).

Table S1. POV characters ranked by number of chapters

Major POV characters		Minor POV characters	
Name	Chapters	Name	Chapters
Tyrion Lannister	47	Asha Greyjoy	4
Jon Snow	42	Victarion Greyjoy	4
Arya Stark	33	Quentyn Martell	4
Daenerys Targaryen	31	Barristan Selmy	4
Catelyn Stark	25	Aeron Greyjoy	2
Sansa Stark	24	Areo Hotah	2
Bran Stark	21	Arianne Martell	2
Jaime Lannister	17	Jon Connington	2
Eddard Stark	15	Arys Oakheart	1
Davos Seaworth	13	Melisandre	1
Theon Greyjoy	13		
Cersei Lannister	12		
Samwell Tarly	10		
Brienne of Tarth	8		

POV characters ranked by number of chapters.

Table S2. Characters ranked by different centrality measures

All characters

Betweenness Centrality	Closeness Centrality	Page Rank	Eigenvector Centrality
1. Jon Snow (0.0889)	1. Jaime Lannister (0.4007)	1. Arya Stark (0.0072)	1. Catelyn Stark (0.1814)
2. Barristan Selmy (0.0831)	2. Tyrion Lannister (0.3943)	2. Jon Snow (0.0067)	2. Jaime Lannister (0.1583)
3. Arya Stark (0.0777)	3. Catelyn Stark (0.3909)	3. Tyrion Lannister (0.0061)	**3. Robb Stark (0.1547)**
4. Tyrion Lannister (0.0700)	4. Eddard Stark (0.3900)	4. Jaime Lannister (0.0060)	4. Tyrion Lannister (0.1451)
5. Theon Greyjoy (0.0671)	**5. Robert Baratheon (0.3895)**	5. Theon Greyjoy (0.0056)	5. Cersei Lannister (0.1392)
6. Jaime Lannister (0.0606)	6. Arya Stark (0.3855)	6. Barristan Selmy (0.0052)	6. Sansa Stark (0.1350)
7. Catelyn Stark (0.0568)	**7. Stannis Baratheon (0.3854)**	7. Catelyn Stark (0.0051)	**7. Tywin Lannister (0.1247)**
**8. Stannis Baratheon (0.0519)**	**8. Joffrey Baratheon (0.3815)**	8. Cersei Lannister (0.0043)	**8. Joffrey Baratheon (0.1198)**
**9. Tywin Lannister (0.0356)**	9. Sansa Stark (0.3804)	**9. Tywin Lannister (0.0042)**	9. Eddard Stark (0.1139)
10. Eddard Stark (0.0351)	10. Jon Snow (0.3755)	10. Eddard Stark (0.0040)	**10. Hosteen Frey (0.1096)**
**11. Robert Baratheon (0.0288)**	11. Cersei Lannister (0.3725)	11. Sansa Stark (0.0040)	**11. Walder Frey (0.1089)**
12. Sansa Stark (0.0275)	**12. Tywin Lannister (0.3720)**	**12. Stannis Baratheon (0.0038)**	**12. Petyr Baelish (0.1079)**
13. Cersei Lannister (0.0250)	**13. Robb Stark (0.3719)**	**13. Robb Stark (0.0038)**	**13. Walder Rivers (0.1068)**
14. Brienne of Tarth (0.0236)	14. Barristan Selmy (0.3702)	14. Daenerys Targaryen (0.0036)	**14. Edmure Tully (0.1055)**
**15. Jorah Mormont (0.0227)**	15. Theon Greyjoy (0.3694)	15. Brienne of Tarth (0.0034)	**15. Tommen Baratheon (0.1027)**
17. Samwell Tarly (0.0207)	17. Bran Stark (0.3640)	18. Samwell Tarly (0.0031)	19. Arya Stark (0.0982)
18. Bran Stark (0.0202)	48. Brienne of Tarth (0.3319)	21. Bran Stark (0.0030)	32. Theon Greyjoy (0.0880)
21. Daenerys Targaryen (0.0185)	76. Samwell Tarly (0.3234)	24. Davos Seaworth (0.0029)	55. Bran Stark (0.0774)
25. Davos Seaworth (0.0167)	166. Davos Seaworth (0.3033)		67. Jon Snow (0.0693)
	227. Daenerys Targaryen (0.2973)		78. Brienne of Tarth (0.0661)
			209. Samwell Tarly (0.0261)
			326. Davos Seaworth (0.0167)
			396. Daenerys Targaryen (0.0125)

Surviving characters only

Betweenness Centrality	Closeness Centrality	Page Rank	Eigenvector Centrality
1. Tyrion Lannister (0.0972)	1. Jaime Lannister (0.3754)	1. Arya Stark (0.0090)	**1. Hosteen Frey (0.1693)**
2. Barristan Selmy (0.0952)	2. Tyrion Lannister (0.3633)	2. Tyrion Lannister (0.0081)	**2. Walder Frey (0.1689)**
3. Arya Stark (0.0923)	3. Arya Stark (0.3617)	3. Jon Snow (0.0078)	**3. Walder Rivers (0.1684)**
4. Theon Greyjoy (0.0909)	**4. Stannis Baratheon (0.3567)**	4. Jaime Lannister (0.0073)	**4. Edwyn Frey (0.1632)**
5. Jon Snow (0.0871)	5. Sansa Stark (0.3545)	5. Theon Greyjoy (0.0065)	**5. Black Walder (0.1595)**
**6. Stannis Baratheon (0.0812)**	6. Theon Greyjoy (0.3453)	6. Barristan Selmy (0.0058)	**6. Lothar Frey (0.1578)**
7. Jaime Lannister (0.0805)	7. Jon Snow (0.3444)	7. Cersei Lannister (0.0054)	**7. Aenys Frey (0.1564)**
8. Sansa Stark (0.0408)	8. Cersei Lannister (0.3429)	8. Sansa Stark (0.0053)	**8. Roslin Frey (0.1560)**
9. Samwell Tarly (0.0320)	9. Bran Stark (0.3407)	**9. Stannis Baratheon (0.0046)**	**9. Fat Walda (0.1496)**
10. Cersei Lannister (0.0310)	**10. Tommen Baratheon (0.3394)**	10. Asha Greyjoy (0.0044)	**10. Ami Frey (0.1470)**
11. Victarion Greyjoy (0.0292)	11. Barristan Selmy (0.3295)	11. Brienne of Tarth (0.0043)	**11. Joyeuse Erenford (0.1465)**
12. Brienne of Tarth (0.0274)	**12. Jeyne Poole (0.3277)**	12. Samwell Tarly (0.0039)	**12. Perwyn Frey (0.1450)**
13. Bran Stark (0.0248)	**13. Roose Bolton (0.3271)**	13. Victarion Greyjoy (0.0039)	**13. Olyvar Frey (0.1435)**
**14. Roose Bolton (0.0212)**	**14. Sandor Clegane (0.3263)**	14. Daenerys Targaryen (0.0036)	**14. Benfrey Frey (0.1393)**
15. Asha Greyjoy (0.0197)	**15. Myrcella Baratheon (0.3219)**	15. Davos Seaworth (0.0036)	**15. Alyx Frey (0.1390)**
17. Davos Seaworth (0.0184)	21. Brienne of Tarth (0.3140)	18. Bran Stark (0.0034)	21. Jaime Lannister (0.1246)
33. Daenerys Targaryen (0.0093)	39. Samwell Tarly (0.3017)		40. Tyrion Lannister (0.0947)
	117. Davos Seaworth (0.2759)		42. Cersei Lannister (0.0916)
	507. Daenerys Targaryen (0.2511)		45. Sansa Stark (0.0866)
			51. Arya Stark (0.0700)
			54. Theon Greyjoy (0.0656)
			58. Brienne of Tarth (0.0604)
			80. Bran Stark (0.0474)
			93. Jon Snow (0.0376)
			193. Samwell Tarly (0.0185)
			247. Davos Seaworth (0.0121)
			424. Daenerys Targaryen (0.0053)

Characters ranked by various importance measures (with values in parentheses). The non-POV characters that appear in the top 15 are highlighted in boldface and major POV characters who do not appear in the top 15 are also listed. Qualitatively it appears that the 14 major POV characters correlate well with the most important characters by all measures.

5. DJ Daley, DV Jones, An Introduction to the Theory of Point Processes: Elementary Theory of Point Processes. (Springer), (2003).
6. M Newman, Networks. (Oxford university press), (2018).
7. A Bavelas, Communication patterns in task-oriented groups. The journal acoustical society Am. **22**, 725–730 (1950).
8. ME Newman, The mathematics of networks in The New Palgrave Encyclopedia of Economics, eds. L Blume, S Durlauf. (Palgrave Macmillan, Basingstoke) Vol. 2, pp. 1–12 (2008).
9. AN Langville, CD Meyer, A survey of eigenvector methods for web information retrieval. SIAM review **47**, 135–161 (2005).
10. L Page, S Brin, R Motwani, T Winograd, The pagerank citation ranking: Bringing order to the web, (Stanford InfoLab), Technical report (1999).
